# Case of Dyspepsia

**Published:** 1817-04

**Authors:** 


*For the London Medical and Physical Journal.*

### *Case of Dyspepsia;* by S. J. E.

MRS. H., widow, aged 38, subject to dyspepsia, was attacked, on October 20th, 1816, with hæmatemesis, from which she recovered in about a fortnight. On the 20th of the following November, she was seized with dyspepsia in its worst form. The symptoms were anorexia, nausea, vomitus, distensions of the stomach, acid eructations for some time after all meals except breakfast, ruminatio, cardialgia, gastrodynia, a sensation of great anxiety referred to the stomach after eating, obstipatio, and, consequently, great debility. For the relief of these symptoms, aromatics, tonics, antispasmodics, aperients, emetics, absorbents, blisters, and a proper attention to diet, had been unsuccessfully resorted to. Imagining that the disease was owing to the acetous fermentation, on February 3d, 1817, I prescribed for her the following:

℞. Potass. Carb. ʒij. Aqua Font. ℥vj. Fiat Mist. cujus Cap. Coch. ij. post mensam singulam.

℞. Infus. Calumbæ ℥iv. Infus. Cascarill. ℥iv. Æther Rectif. ʒiij. Fiat Mist. cujus Cap. Coch. ij. bis terve die.

℞. Potass. Sulph. ʒſs. Pulv. Rhei. ℈j. Aqua Menth. p. ℥j. Misce fiat Haust. cras mane sumendus, et repetandus p. r. n.

The alkaline solution produced a copious eructation of carbonic acid gas, and a great secretion of urine,—which was a sufficient proof of its being decomposed in the stomach by acetic acid, forming the acetate of potass. The other mixture removed the spasm, and gave tone to the debilitated coats of the stomach. As the strength of the stomach increased, the tendency to generate acetic acid was diminished; for, after she had taken the solution of carbonate of potass eight days, she felt no pain in the region of her stomach; and, on recurring to it again, it produced nausea, with no eructations of carbonic acid gas;—it was consequently discontinued. She took the antispasmodic mixture another week, at the end of which she was perfectly recovered.

As frequent obstinate cases of dyspepsia are met with, in general, probably owing to the debilitated stomach, producing the acetous fermentation on the food, would not this be an improved method of treatment?

*March* 4, 1817.

**P.S.—S. J. E. must solicit pardon for the apparent inattention to**

to his promise in No. 201 of this Journal; and likewise gratefully assure E. C. B. that his vanity was flattered, and his medical knowledge not a little improved, by his learned, judicious, and friendly lesson in No. 203; assuring him, that the treatment there alluded to was *approved of* and adopted, but has hitherto *proved* unsuccessful.

